# Using fluorescently labeled wheat germ agglutinin to track lipopolysaccharide transport to the outer membrane in *Escherichia coli*

**DOI:** 10.1128/mbio.03950-24

**Published:** 2025-02-24

**Authors:** Laurent Dubois, Andrea Vettiger, Jackson A. Buss, Thomas G. Bernhardt

**Affiliations:** 1 Department of Microbiology, Harvard Medical School, Boston, Massachusetts, USA; 2 Howard Hughes Medical Institute, Chevy Chase, Maryland, USA; Indiana University Bloomington, Bloomington, Indiana, USA

**Keywords:** lipopolysaccharide, wheat germ agglutinin, outer membrane

## Abstract

The cell envelope of gram-negative bacteria consists of two membranes sandwiching the peptidoglycan (PG) cell wall. The outer membrane (OM) contains integrated beta-barrel proteins and has an outer leaflet composed of lipopolysaccharide (LPS). LPS is transported from the inner membrane where it is made to the OM surface by the Lpt system. In the polarly elongating alpha-proteobacterium *Brucella abortus*, LPS transport has been localized to the polar growth zone and division site. However, LPS transport has not been tracked in live proteobacteria like *Escherichia coli* that elongate by dispersed incorporation of envelope material along their cell body. Here, we report an investigation into the binding target of fluorescently labeled wheat germ agglutinin (FL-WGA) on *E. coli* cells that led to the development of a method for visualizing LPS transport. We show that instead of PG or enterobacterial common antigen for which FL-WGA labeling has been used to detect in the past, this probe recognizes LPS modified with a terminal N-acetylglucosamine formed by the defective O-antigen synthesis pathway of laboratory strains of *E. coli*. This finding enabled the construction of mutants inducible for LPS modification that were used together with FL-WGA labeling to track LPS transport to the cell surface. We show that new LPS is inserted throughout the cell cylinder and at the division site, but not at the cell poles. A similar pattern was observed previously for PG synthesis and OM protein insertion in *E. coli*, suggesting that LPS transport to the OM is coordinated with these processes.

**IMPORTANCE:**

Gram-negative bacteria like *Escherichia coli* are surrounded by a multilayered cell envelope that includes an outer membrane (OM) responsible for their high intrinsic resistance to antibiotics. The outer leaflet of this membrane is composed of a glycolipid called lipopolysaccharide (LPS). Here, we report the development of an imaging method to track the transport of LPS to the *E. coli* outer membrane. The results indicate that transport occurs throughout the cell cylinder and at the division site, but not at the cell poles. A similar pattern was observed previously when cell wall synthesis and the insertion of proteins into the OM were tracked. Our results therefore suggest that LPS transport to the OM is coordinated with other essential processes that underly gram-negative cell envelope biogenesis.

## INTRODUCTION

Most bacteria are surrounded by a complex cell envelope that is essential for cell shape and integrity. The envelope of gram-negative bacteria like the model gamma-proteobacterium *Escherichia coli* consists of two membranes forming an intermembrane aqueous compartment called the periplasm where the peptidoglycan (PG) cell wall is built (1, 2). This envelope architecture is a major determinant of the intrinsic drug resistance of gram-negative bacteria (2, 3), and the assembly pathways that construct it are the targets of many of our most effective antibiotic treatments (4). Therefore, in addition to being fundamentally important for understanding how bacterial cells grow, elucidating the mechanism underlying gram-negative envelope biogenesis has practical implications for the development of new treatments for antibiotic-resistant bacterial infections.

The permeability barrier that blocks the entry of many drugs into gram-negative bacteria is formed primarily by the outer membrane (OM) layer of the envelope (2, 3). This unique membrane contains islands of beta-barrel outer membrane proteins (OMPs) and an asymmetric lipid bilayer with phospholipids in the inner leaflet and the glycolipid lipopolysaccharide (LPS) in the outer leaflet (2, 3). Although LPS has a relatively conserved core structure, there are important features that vary between bacteria. All forms consist of a lipid A base made from a disaccharide of glucosamine modified with multiple acyl chains and a core oligosaccharide (5). LPS without further modification to the core oligosaccharide is commonly referred to as “rough” LPS or as lipooligosaccharide. In many gram-negative bacteria, the rough LPS is further modified by the addition of a long glycopolymer called O-antigen (O-Ag) (6). In enterobacteria like *Escherichia coli*, the core LPS is also modified by another glycan referred to as enterobacterial common antigen (ECA) (7). As its name implies, the structure of ECA is conserved between different strains and species whereas the composition of the O-Ag polymer is variable from organism to organism. In this report, we use the designations ^O-Ag^LPS and ^ECA^LPS to refer to core/rough LPS modified with O-Ag or ECA, respectively.

Whether it is modified or not, LPS must be transported from the inner (cytoplasmic) membrane where it is synthesized to the outer leaflet of the OM (8). This transport process is mediated by the conserved Lpt transport system (9–16). An ATP-binding cassette transporter formed by LptB, LptF, and LptG extracts LPS from the outer leaflet of the inner membrane and transfers it to the associated LptC component of the inner membrane complex (17). The periplasmic LptA component connects LptC in the inner membrane complex with the OM components LptD and LptE, forming a transenvelope bridge on which LPS traverses to reach the outer leaflet of the OM (18–20). In the pathogenic alpha-proteobacterium *Brucella abortus* that elongates via unipolar extension (21), components of the Lpt system localize to the growth pole and the cell division site where new LPS has been observed to be transported to the cell surface (22, 23). Unlike *B. abortus* and some other alpha-proteobacteria like *Agrobacterium tumefaciens* that grow by tip extension, *E. coli* and related gamma-proteobacteria elongate by inserting new envelope material at dispersed locations throughout their cell body (24–28). Although single-molecule experiments have detected Lpt bridge formation in *E. coli* (20), the location of bulk LPS transport in live cells of this model gamma-proteobacterium has remained unclear.

In this report, we describe the serendipitous discovery of a method to track LPS transport in live *E. coli* using fluorescently labeled wheat germ agglutinin (FL-WGA). We show that instead of PG or ECA that have been proposed to be the target of FL-WGA (29, 30), this probe recognizes LPS modified with a terminal N-acetylglucosamine formed by the defective O-antigen synthesis pathway of laboratory strains of *E. coli* (31, 32). This finding enabled the construction of mutants inducible for LPS modification that were used together with FL-WGA labeling to track LPS transport to the cell surface. We show that new LPS is inserted throughout the cell cylinder and at the division site, but not at the cell poles. A similar pattern was observed previously for PG synthesis and OM protein insertion in *E. coli* (26–28, 33–35), suggesting that LPS transport to the OM is coordinated with these processes in bacteria that grow by a dispersed mode of cell elongation as well as those that grow by polar extension.

## RESULTS

### PG is unlikely to be the binding substrate of FL-WGA in *E. coli*

**Fig 1:**
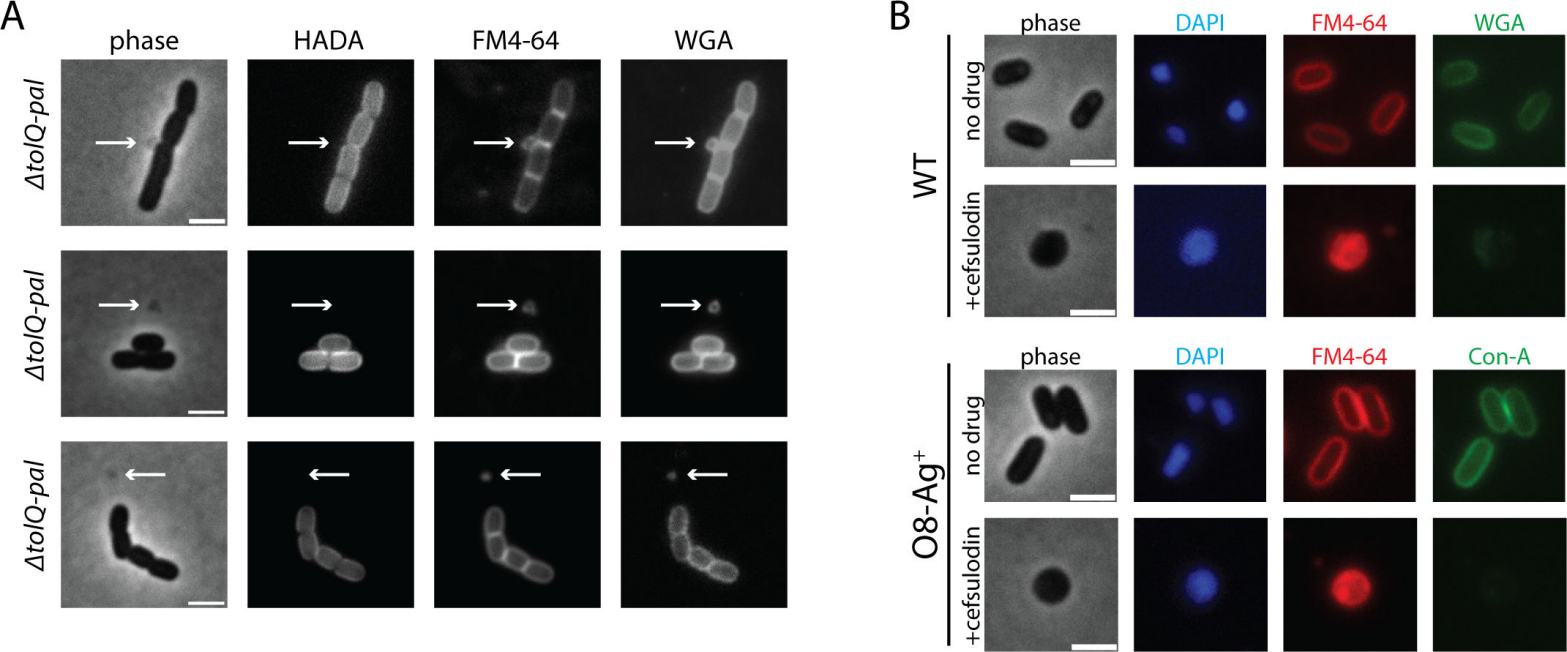
Evidence that FL-WGA binds a molecule on the OM surface. (A) Cells of strain MT91 [Δ*tol-pal*] were stained with the indicated dyes and imaged using phase and the appropriate fluorescence optics. Bar equals 2 µm. (B) Cultures of MG1655 [WT] or strain JAB595 [*rfb*O8, O8-Ag^+^] were grown with or without cefsulodin to induce the formation of L-form-like cells. They were then stained with the indicated dyes and imaged using phase and the appropriate fluorescence optics. See Materials and Methods for details. Bar equals 2 µm.

Wheat germ agglutinin (WGA) is a lectin that is known to bind N-acetylglucosamine (GlcNAc) (36, 37). Because the PG cell wall contains GlcNAc, FL-WGA derivatives have been used previously for *E. coli* imaging experiments with the goal of labeling the PG cell wall (29). However, given that WGA forms a 38 kDa dimer (36), it is unlikely to cross the OM to reach the PG layer. Instead, a molecule exposed on the outer surface of the OM is a more plausible binding substrate for FL-WGA. To investigate this possibility, we first took advantage of the phenotype of mutants defective for the Tol-Pal system (38). Cells lacking this system form cell chains that produce OM vesicles and blebs that typically emanate from division sites due to poor anchoring of the OM to the PG layer during cytokinesis (39). When these mutants were treated with FL-WGA, the labeling pattern mirrored that of the membrane dye FM4-64 with the fluorescence signal concentrated around the cell periphery and at division septa, including labeling of OM blebs and vesicles (Fig. 1A). However, the OM blebs/vesicles were not labeled with the fluorescent D-amino acid HADA that is incorporated into the PG layer (Fig. 1A) (28). The differential labeling of OM blebs and vesicles with FL-WGA and HADA suggests that FL-WGA is not binding PG.

In addition to mutants defective for the Tol-Pal system, we also investigated the binding of fluorescent lectins to L-form-like *E. coli* cells that have greatly reduced levels of PG (40). These L-form-like cells are formed by treating cultures with the beta-lactam cefsulodin in the presence of sucrose for osmotic stabilization (40). Prior work showed that such L-forms do not bind FL-WGA, a result used to support the idea that the probe is recognizing PG (29). However, an alternative interpretation of this result is that disruption of PG synthesis by cefsulodin disrupts formation of the FL-WGA target on the OM. To test this possibility, we repeated the L-form binding experiments with wild-type *E. coli* and a derivative producing a heterologous O8-type O-Ag (41). The O8-type O-Ag is a binding substrate for fluorescently labeled derivatives of the lectin concanavalin A (FL-ConA) (41). This probe does not label cells lacking the O8 O-Ag and thus does not bind PG (41). As observed previously (29), we found that FL-WGA poorly labels cefsulodin-induced L-forms of wild-type *E. coli* (Fig. 1B; Fig. S1). Notably, cefsulodin treatment also blocked FL-ConA labeling of cells with the O8-type O-Ag (Fig. 1B; Fig.S1). This result indicates that cefsulodin-induced L-forms have a defect that generally interferes with OM formation and/or LPS modification in addition to the near total loss of PG. Thus, the lack of FL-WGA binding to cefsulodin-induced L-forms of wild-type *E. coli* does not unequivocally support PG as a binding substrate for FL-WGA. Furthermore, wild-type (WT) cells permeabilized by treatment with sodium dodecyl sulfate (SDS) and ethylenediaminetetraacetic acid (EDTA) did not label with FL-WGA (Fig. S2). These cells have likely been stripped of their OM by this treatment but retain the PG layer as indicated by HADA labeling (Fig. S2). Based on this result and the results with the *tol-pal* mutants and the L-forms, we conclude that FL-WGA is most likely binding a target on the OM surface, not the PG layer in the periplasm of *E. coli* as has been previously proposed (29). Accordingly, prior results in gram-positive bacteria also indicate that FL-WGA does not bind PG in these organisms for which it is also often used as a probe (42–44, e.g.,), but rather it binds GlcNAc-modified teichoic acids (45–47).

### Evidence that FL-WGA is not recognizing ^ECA^LPS

**Fig 2:**
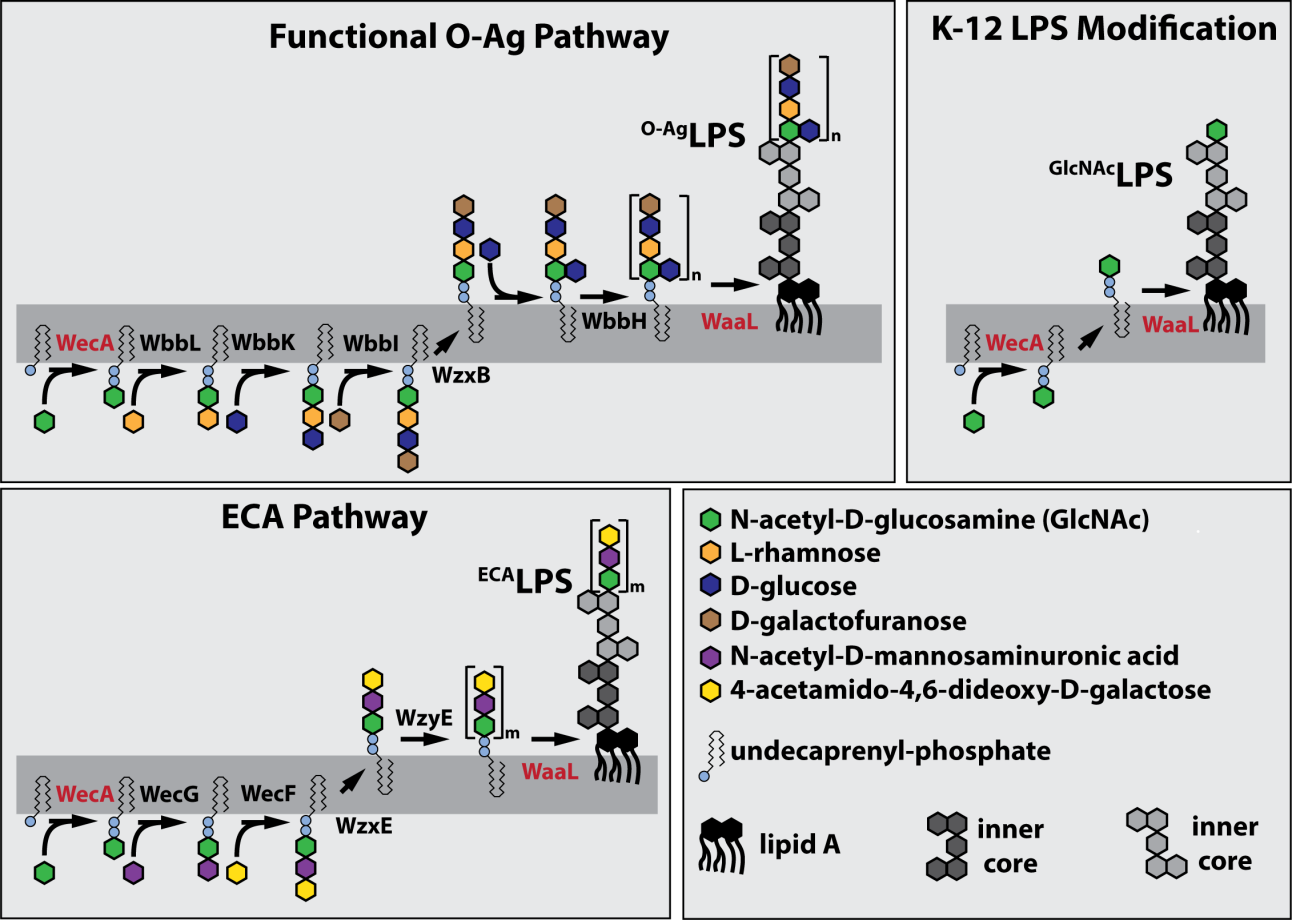
Pathways for LPS modification in *E. coli*. Shown are simplified diagrams of the ^O-Ag^LPS, ^ECA^LPS, and ^GlcNAc^LPS synthesis pathways. For simplicity, the activating nucleotide for the sugar subunits are not shown in the diagram. Also, the enzymatic steps involving the synthesis of different activated sugars or sugar modifications are not shown. See text for details.

Because they contain a GlcNAc sugar in their repeating units, alternative candidates for the binding substrate of FL-WGA are the surface-exposed O-Ag (O16-type) and ECA polymers attached to the LPS of *E. coli*. The synthesis pathways of both O-Ag and ECA begin at the inner surface of the inner membrane with the transfer of phospho-GlcNAc from UDP-GlcNAc to the lipid carrier undecaprenyl-phosphate by WecA to form the lipid I precursor of O-Ag and ECA (^ECA/O-Ag^lipid I) (Fig. 2) (7, 48–51). The pathways then diverge with different sugars being added to ^ECA/O-Ag^lipid I to form the final lipid-linked precursor of the respective polymers. These building blocks are then flipped by Wzx/MOPS-type transporters to expose the sugar headgroups on the outer surface of the inner membrane where they are then polymerized into O-Ag or ECA, transferred to LPS by the ligase WaaL, and transported to the OM by the Lpt system (Fig. 2) (6, 7, 9). Importantly, K-12 strains of *E. coli* like the one we use here and that are commonly used in molecular biology laboratories are defective for O-Ag synthesis (31). An insertion sequence interrupts their *wbbL* gene encoding the enzyme catalyzing the second step in the O-Ag synthesis pathway (31). We therefore initially thought that ECA was likely to be the binding site of FL-WGA as has previously been proposed in studies that have used FL-WGA as a probe for surface-exposed ECA (30).

**Fig 3:**
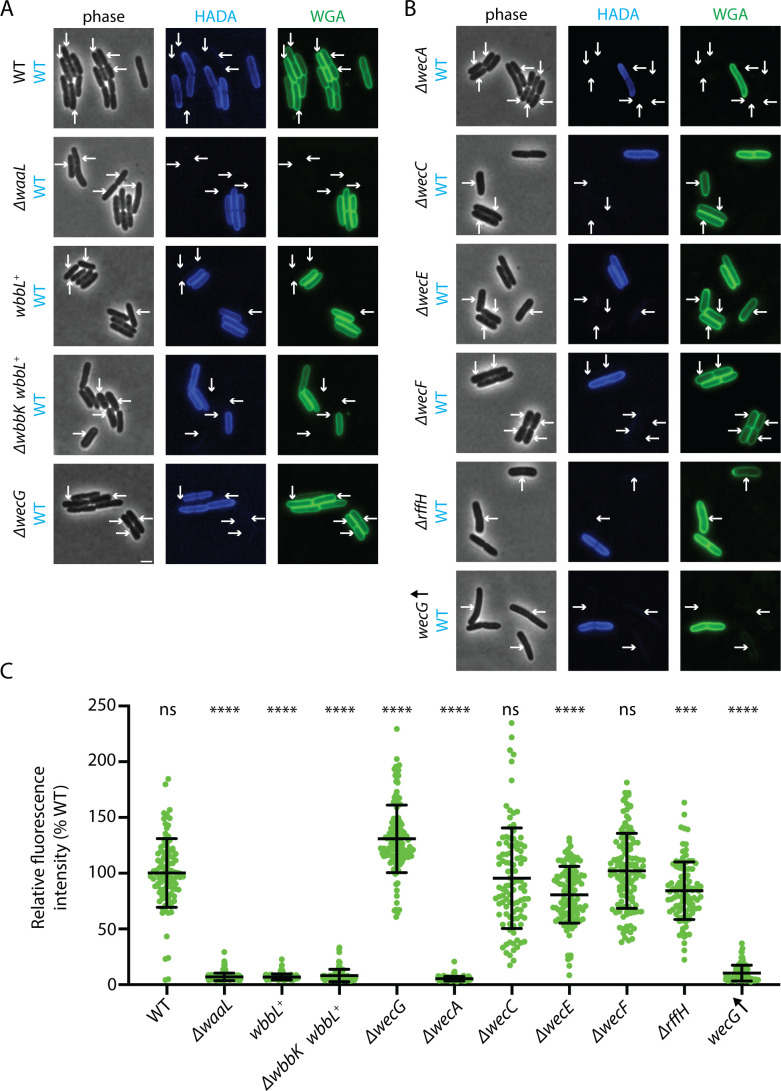
FL-WGA labels specifically ^GlcNAc^LPS. Strains with mutations in genes in the O-Ag (**A**) or ECA (**B**) pathways were mixed with a roughly equal amount of HADA-labeled MG1655 [WT] cells. The mixtures were then labeled with FL-WGA and imaged using phase and the indicated fluorescence optics. Bar equals 2 µm. In each panel, the arrows point to mutant cells identified by their failure to label with HADA. (C) The intensity of FL-WGA labeling of the indicated mutant cells was compared to the mean labeling intensity of WT cells in the same mixture (identified by HADA labeling) and represented as the percentage of WT labeling. For each condition, at least 100 cells were analyzed. The *P* value was calculated using unpaired *t*-test with Welch’s correction; ns, not significant; ***P <0.001; ****P <0.0001. The average fluorescence intensity was as follows: MG1655 [WT] = 100.2% (*P* value = 0.9518), LD137 [Δ*waaL*] = 6.983% (*P* value < 0.0001), LD189 [*wbbL*^+^] = 6.920% (*P* value < 0.0001), LD208 [Δ*wbbK wbbL*^+^] = 8.230% (*P* value < 0.0001), LD159 [Δ*wecG*] = 130.8% (*P* value < 0.0001), LD177 [Δ*wecA*] = 5.332 (*P* value < 0.0001), LD149 [Δ*wecC*] = 95.49 (*P* value = 0.4320), LD142 [Δ*wecE*] = 80.66 (*P* value < 0.0001), LD146 [Δ*wecF*] = 102.1 (*P* value = 0.6431), LD144 [Δ*rffH*] = 84.33 (*P* value = 0.0003), and LD502 [wecG↑] = 10.39 (*P* value < 0.0001).

To test the ECA requirement for FL-WGA labeling of *E. coli*, we mixed samples of HADA-labeled WT cells with unlabeled cells of various genetic backgrounds before exposing them to FL-WGA. In control mixtures containing both HADA-labeled and unlabeled WT cells, all cells in the field identified by phase-contrast imaging were stained with FL-WGA regardless of their HADA-labeling status (Fig. 3). By contrast, when HADA-labeled WT cells were mixed with unlabeled Δ*waaL* mutant cells defective for the O-Ag/ECA ligase, only the HADA-positive WT cells were stained with FL-WGA (Fig. 3). Thus, consistent with ECA being the binding substrate, the WaaL ligase is required for FL-WGA labeling as previously reported (30). Unexpectedly, however, cells of a Δ*wecG* mutant defective for the second enzymatic step in the ECA pathway (Fig. 2) were found to label with FL-WGA even better than WT cells (Fig. 3). Indeed, of all the ECA synthesis pathway mutants tested, the only other mutant that failed to label with FL-WGA was Δ*wecA*, which is inactive for making ^ECA/O-Ag^lipid I (Fig. 3). We therefore conclude that ECA is not the binding substrate of FL-WGA recognized during cell labeling.

### LPS modified by a single GlcNAc is the likely binding target of FL-WGA

If FL-WGA is not recognizing ^ECA^LPS, then what might it be recognizing? Based on the WecA and WaaL requirements for FL-WGA labeling, we hypothesized that ^ECA/O-Ag^lipid I may be the source of the LPS modification recognized by FL-WGA. We suspected that a fraction of ^ECA/O-Ag^lipid I produced in WT cells might be being flipped and used as a substrate of WaaL to transfer a single GlcNAc sugar to the end of the outer core oligosaccharide, forming a molecule we refer to as ^GlcNAc^LPS (Fig. 2). Indeed, ^GlcNAc^LPS has been detected by mass spectrometry and in blots of LPS preparations from *E. coli* K-12 using WGA as a probe, and the formation of this molecule was found to depend on WecA and the Wzx flippase (32). Evidence was also presented that the GlcNAc sugar needed to be the terminal sugar on LPS to be recognized by WGA (32). Accordingly, cells restored for O-Ag synthesis by complementation of the WbbL defect failed to stain with FL-WGA (Fig. 3). Furthermore, a strain restored for WbbL activity but inactivated for the third enzyme in the O-Ag synthesis pathway (WbbK) also failed to label with FL-WGA (Fig. 3). In this strain, the LPS is predicted to be modified with both GlcNAc and rhamnose (Fig. 2) such that the GlcNAc is no longer the terminal sugar modifying the core LPS. Importantly, these strains that fail to label with FL-WGA remain capable of making ECA. Moreover, overexpression of *wecG* to potentially enhance ECA synthesis resulted in reduced FL-WGA labeling (Fig. 3). Together with the observation that Δ*wecG* labels more robustly than WT cells, these results suggest that the synthesis of ^ECA^LPS competes with ^GlcNAc^LPS production and reduces FL-WGA labeling. We conclude that ^GlcNAc^LPS is the most likely binding substrate of FL-WGA, not PG or ECA for which this lectin has previously been used as a probe in *E. coli* labeling experiments (29, 30).

### Using FL-WGA to track LPS transport

**Fig 4:**
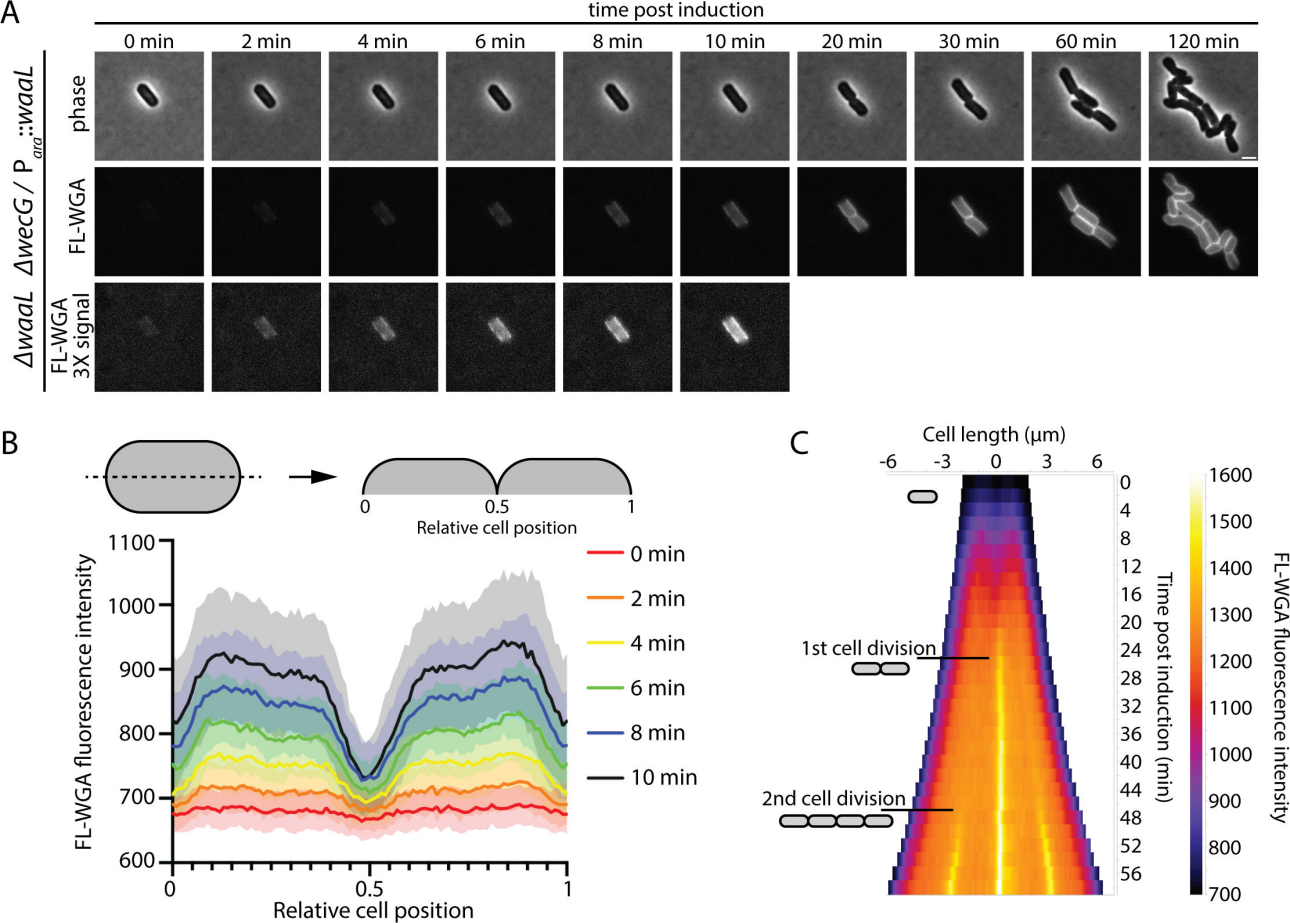
Tracking LPS transport with an inducible *waaL* strain. (A) Representative images of LD228 [Δ*waaL* Δ*wecG*/*P_ara_::waaL*] cells following the induction of *waaL* expression with arabinose and labeling with FL-WGA. Bar equals 2 µm. (B) FL-WGA fluorescence intensity was quantified around the cell contour at the indicated time points. Positions 0, 0.5, and 1 are at the cell poles as shown in the diagram above the graph. At least 70 cells were analyzed per time point. (C) Representative kymograph of FL-WGA labeling of a single cell over the course of two cell doublings following induction of *waaL*. Note that the original poles remain unlabeled throughout the time course.

The finding that FL-WGA recognizes ^GlcNAc^LPS on the cell surface suggested a method for using the labeled lectin to track the spatiotemporal dynamics of LPS transport to the OM. To this end, a strain was constructed with deletions of *wecG* and *waaL* to block the conversion of ^ECA/O-Ag^lipid I to downstream ECA precursors and the transfer of GlcNAc to LPS, respectively. A plasmid encoding *waaL* under control of the arabinose promoter (P*_ara_::waaL*) was then introduced to allow for the induction of ^GlcNAc^LPS formation upon the addition of arabinose to the growth medium. To follow LPS transport, the labeling strain was grown to mid-exponential phase in lysogeny broth (LB) lacking inducer. Cells were then deposited on LB agarose pads supplemented with FL-WGA and arabinose and imaged by time-lapse fluorescence microscopy. At early time points following induction, FL-WGA labeling was detected throughout the cell cylinder of newborn cells with an intensity that increased over time (Fig. 4A through C). This result is consistent with prior electron microscopy studies that tracked LPS transport in *Salmonella*, showing islands of new LPS appearing at dispersed sites on the cell cylinder (52). When cell constriction was initiated at midcell, labeling was detected within the developing septum, which upon further growth gave rise to labeled daughter cell poles (Fig. 4A through C). FL-WGA labeling was never detected at the old cell poles even after several generations of growth in the presence of FL-WGA (Fig. 4A through C). This general labeling pattern was observable in time-lapse micrographs of single cells and corresponding kymographs of single cells that show the quantification of labeling intensity over time (Fig. 4A and C). Additionally, analysis of fluorescence intensity around the periphery of cells in a population at several time points following *waaL* induction similarly showed that labeling was initially restricted to the cell body and no labeling was detected at the old cell poles (Fig. 4B). Thus, the transport of new LPS appears to be restricted to areas along the growing cell cylinder and the division site with transport to the poles only taking place as they are formed during cytokinesis.

**Fig 5:**
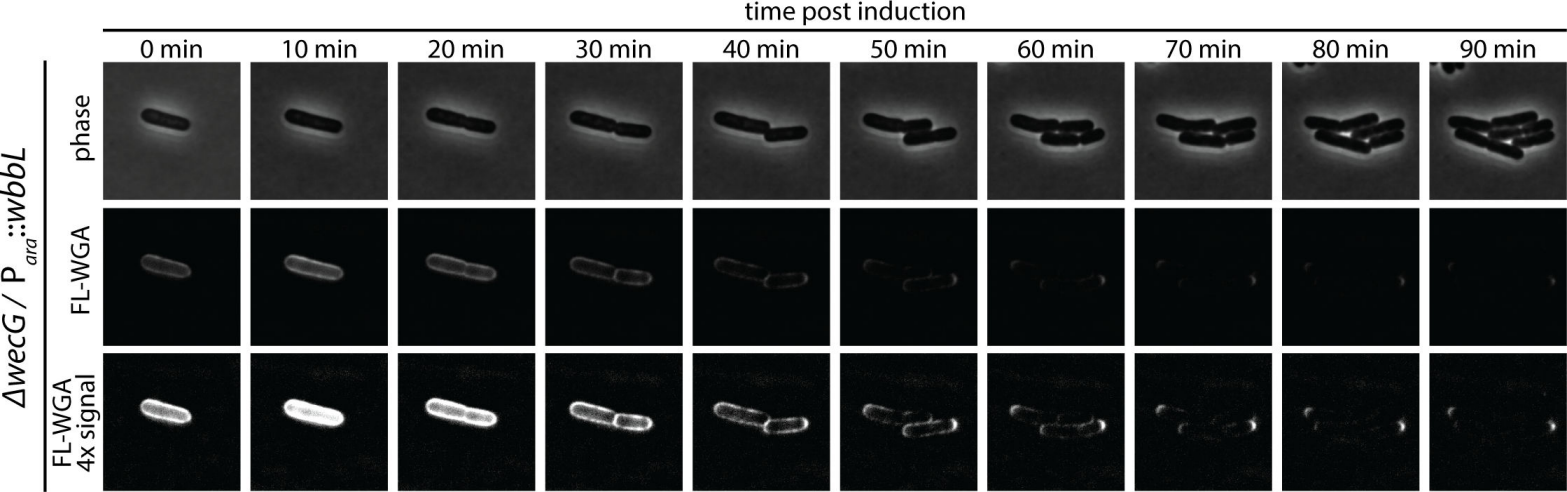
An alternative approach for tracking LPS transport. Representative images of LD206 [Δ*wecG*/*P_ara_::wbbL*] following the induction of O antigen synthesis by the addition of arabinose and FL-WGA labeling. Note that in this experiment, cells start out being completely labeled because ^GlcNAc^LPS synthesis is functional at the outset. However, upon induction of O-Ag synthesis, FL-WGA labeling is blocked in new growth zones. The old poles remain labeled for at least two doublings, indicating that there is no new LPS transport at the cell poles.

**Fig 6:**
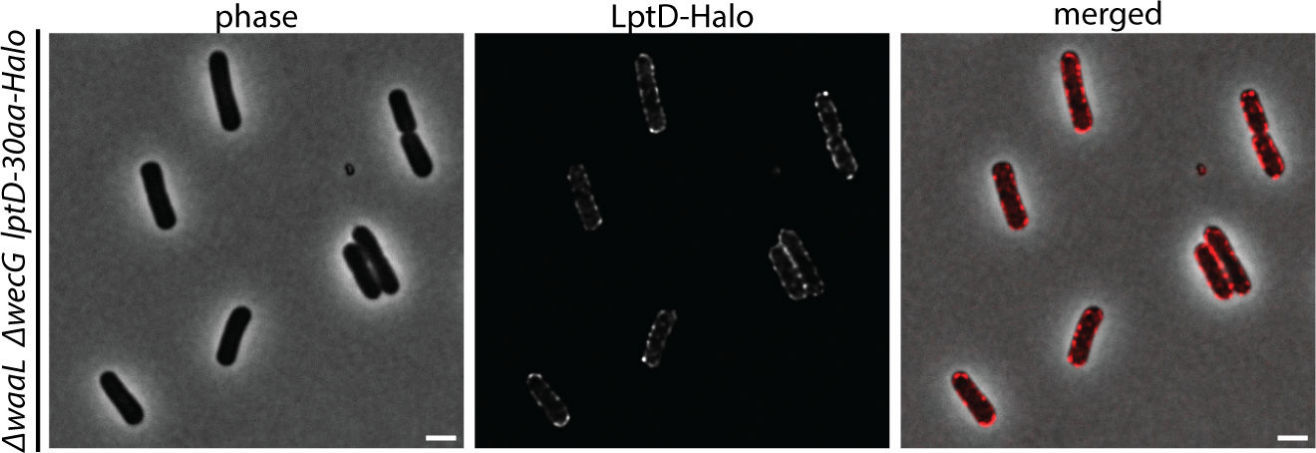
LptD is distributed around the cell periphery. Shown are representative cells of LD338 [*lptD-halo*] labeled with the Halo-ligand JF646 and imaged using phase and fluorescence optics.

To validate the labeling results based on the induction of ^GlcNAc^LPS production, we used an alternative method for tracking LPS transport based on the restoration of ^O-Ag^LPS production. We reasoned that restoring O-Ag synthesis would block ^GlcNAc^LPS formation such that new LPS produced following the restoration would be unable to bind FL-WGA. Thus, in this case, LPS transport could be detected by the loss of labeling signal. For this method, a plasmid harboring *wbbL* under P_ara_ control was introduced to a *wecG* deletion strain that like other K-12 strains is defective for the WbbL-catalyzed step of the O-Ag synthesis pathway (31). A culture of this strain was then deposited on LB agarose pads supplemented with FL-WGA and arabinose for imaging (Fig. 5). Because the cells were constitutively producing ^GlcNAc^LPS prior to the induction of *wbbL* expression, they initially displayed a FL-WGA signal around their entire periphery. Over time, this signal was gradually depleted in the cell body, indicative of new, O-Ag modified LPS transport throughout the cell cylinder (Fig. 5). However, the old poles remained labeled for several generations, demonstrating that LPS transport does not occur at the poles once they are formed (Fig. 5). Notably, localization of LptD in cells producing a functional LptD-Halo fusion (19) showed that the OM component of the LPS transporter remains present at the cell poles despite the lack of LPS transport at these sites (Fig. 6). These results confirm the pattern of LPS transport observed using the inducible production of ^GlcNAc^LPS and suggest that Lpt bridge formation is spatially controlled in cells to coordinate LPS transport with other major cell envelope assembly processes.

## DISCUSSION

FL-WGA is commonly used as a general stain to label bacteria for fluorescence imaging. However, it is often unclear what specific molecule(s) the lectin is recognizing in cells, especially for *E. coli* for which FL-WGA has been employed as a label for molecules as disparate as PG and ^ECA^LPS (29, 30). Our genetic and imaging results indicate that neither of these molecules is likely to be the binding substrate for FL-WGA in *E. coli*. Instead, our findings are most consistent with FL-WGA binding to LPS modified by a single GlcNAc sugar (^GlcNAc^LPS) that is formed by the defective O-Ag synthesis pathway present in K-12 strains (32). Using this new information, we generated strains where FL-WGA labeling could be switched on or off as a means to track the spatiotemporal localization of LPS transport in cells based on the pattern of FL-WGA labeling. These imaging experiments revealed that ^GlcNAc^LPS transport occurs throughout the cell body during cell elongation and at midcell during division but never at the cell poles once they are formed.

It is possible that the transport of ^GlcNAc^LPS detected by FL-WGA reflects the transport of this specific molecule and not general LPS transport. However, the entire periphery of wild-type cells is labeled with FL-WGA, indicating that ^GlcNAc^LPS is evenly distributed around the OM and not localized to specialized regions of the membrane. Additionally, both the positive labeling method employing *waaL* induction to initiate ^GlcNAc^LPS production and the negative labeling method involving the restoration of O-Ag synthesis to block FL-WGA binding suggested a similar pattern of LPS transport. Finally, given that the interaction of LPS with the Lpt transport system is largely mediated by the lipid A portion of the molecule (9), the transporter is unlikely to distinguish between LPS molecules with different modifications attached to the outer core, which is located some distance from lipid A. We therefore conclude that FL-WGA labeling upon the induction of ^GlcNAc^LPS production provides an accurate picture of the spatiotemporal dynamics of general LPS transport in *E. coli* during cell growth.

It has been appreciated for some time that diffusion of LPS and OMPs in the OM is limited (34, 35, 41, 53, 54). Expansion of the OM is thus restricted to sites of LPS and/or OMP insertion just as the growth of the PG layer occurs only where new material is added to the matrix by PG synthases. Cells are therefore likely to tightly coordinate OM and PG growth to ensure uniform expansion of these outer envelope layers. Indeed, recent work has shown that areas of active OMP insertion by the Bam machinery in *E. coli* coincide with locations of PG synthesis (55). The pattern of LPS transport we observe mirrors those detected previously for *E. coli* PG synthesis and OMP insertion into the OM (26–28, 33–35). Accordingly, the prior results of Ursell et al. that used FL-WGA to track cell wall growth showed a strong correlation between growth zones detected using FL-WGA with those detected using FDAA labeling and sites of MreB localization (29). Reinterpretation of their findings in light of our new results suggests that sites of LPS transport occur in areas of active PG synthesis. We therefore propose that LPS transport is coordinated with PG synthesis and OMP transport in *E. coli*.

Our findings add to the growing body of evidence indicating a coordination of LPS transport with areas of PG expansion. In the tip-growing alpha-proteobacterium *B. abortus*, LPS transport was shown to occur at the pole and the division site where PG synthesis and OMP insertion are also localized (22, 23). Additionally, in another alpha-proteobacterium, *Caulobacter crescentus*, the PG layer is elongated zonally from midcell for a significant fraction of the cell cycle (56). New S-layer proteins have recently been observed to be inserted into the S-layer in these areas of PG expansion (57). The S-layer is a proteinaceous layer forming an outer shell over the LPS of the OM. Areas of S-layer protein insertion are therefore likely to correspond to areas of OM expansion and LPS transport such that the localization studies of S-layer insertion also support a coordination of LPS transport with PG synthesis.

Although the mechanism connecting LPS transport with PG expansion remains to be determined, it likely involves the regulation of Lpt bridge formation. Our results and those in *B. abortus* (22) indicate that the OM component of the bridge, LptD, is present throughout the cell periphery encompassing areas of both active and inactive LPS transport. Thus, docking points in the OM for bridge formation do not appear to be limited. Instead, formation of the connection between LptD and the inner membrane Lpt components appears to be the control step. Accordingly, in both *B. abortus* and its polar-growing relative *Agrobacterium tumefaciens*, inner membrane components of the Lpt machinery are enriched at the elongating cell pole where LPS transport and PG expansion are occurring (22). An important future direction is therefore to determine the factors responsible for promoting Lpt bridge formation in areas of active PG growth or vice versa given that the two processes might be connected via a mutually reinforcing cycle that may also involve OMP insertion.

In conclusion, we have determined the binding substrate for WGA on surface of *E. coli* cells and used this information to develop a method for tracking LPS transport. Our results reveal that LPS transport occurs in areas known to be active for PG synthesis and OMP insertion into the OM, indicating that these processes are likely to be coordinated in bacteria that grow via a dispersed elongation mechanism as well as in organisms that elongate from their cell pole.

## MATERIALS AND METHODS

### Bacterial strains and growth conditions

All strains used in this study are derivatives of MG1655 (18). They are listed in Table S1. Unless otherwise indicated, cultures were grown at 30°C in LB broth (1% tryptone, 0.5% yeast extract, 0.5% NaCl) or M9 minimal medium (58) supplemented with 0.2% glucose and 0.2% casamino acids. For selection, antibiotics were used at the following concentrations: 25 µg/mL kanamycin (Kan) and 10 µg/mL tetracycline (Tet). Phage P1 transduction (58) was performed to move mutant alleles between the strains. The Kan^R^ cassette used was from pKD13 (59). It was removed from strains as needed using FLP recombinase expressed from pCP20 (59). All mutations were confirmed by diagnostic PCR.

### Imaging Δtol-pal cells

Overnight cultures were grown in LB at 30°C. The next day, they were back diluted 1:100 into 5 mL M9 supplemented with 0.2% glucose and 0.2% casamino acids. Cultures were grown to an OD_600_ of 0.2 to 0.4. At this point, 10 µg/mL Alexa-488-WGA (FL-WGA) and 100 µM HADA were added to the cells and incubated in the dark on a roller at room temperature for 30 min. The cells were collected by centrifugation (2 min, 4,000 × *g*, RT) and washed twice in M9 supplemented with 0.2% glucose. After the last wash, the cells were concentrated 10× and imaged on a 1% agarose pad containing M9 medium (0.2% glucose) supplemented with 5 µg/mL FM4-64 (T3166, Thermo Fisher Scientific, MA, USA), covered with a #1.5 coverslip, and imaged as described below.

### L-form preparation

Overnight cultures were grown in LB at 30°C. The next day, they were back diluted 1:100 into 5 mL M9 medium supplemented with 0.2% glucose, 0.2% casamino acids, and 4% sucrose. Cultures were grown for 2 h at 37°C. At this point, 200 µg/mL cefsulodin was added, and the cells were transferred to 30°C, where they were grown overnight (~20 h). The next day, the cells were incubated for 1 h at room temperature (RT) with 10 µg/mL DAPI (D9542, Sigma-Aldrich, MO, USA) and either 10 µg/mL Alexa-488-WGA (FL-WGA) or 200 µg/mL Alexa-488-concanavalin A (FL-Con-A) (C11252, Thermo Fisher Scientific, MA, USA). The cells were collected by centrifugation (2 min, 4,000 × *g*, RT) and washed twice in M9 medium supplemented with 0.2% glucose and 4% sucrose. After the last wash, the cells were concentrated 20× and spotted on a 1% agarose pad containing M9 medium (0.2% glucose, 4% sucrose) supplemented with 5 µg/mL FM4-64 (T3166, Thermo Fisher Scientific, MA, USA), covered with a #1.5 coverslip, and imaged as described below.

### Labeling cells with FL-WGA to identify the binding target

Overnight cultures were grown in LB supplemented with 0.2% glucose and the appropriate antibiotic at 30°C. The next day, the cultures were diluted 1:100 into LB supplemented with 0.2% arabinose and the appropriate antibiotic, and cells were grown until they reached an OD_600_ of 0.2 to 0.4. MG1655 [WT] was then incubated with 100 µM HADA at 30°C. After 30 min, MG1655 was harvested by centrifugation (2 min, 5,000 × *g*, RT) and washed twice with LB supplemented with 0.2% arabinose. MG1655 was then mixed 1:1 with the ECA and LPS mutants. The mixed cells were harvested by centrifugation (2 min, 5,000 × *g*, RT) and concentrated 10× in LB supplemented with 0.2% arabinose and 10 µg/mL Alexa-594-WGA. The cells were immediately spotted on a 1% agarose pad containing LB medium supplemented with 0.2% arabinose and 10 µg/mL Alexa-594-WGA, covered with a #1.5 coverslip, and imaged as described below.

### Labeling cells treated with SDS and EDTA

Overnight cultures of MG1655 were grown in LB at 30°C. The next day, the cells were back diluted 1:100 into LB and grown at 30°C until they reached an OD_600_ of 0.2 to 0.4. Then, 5% SDS, 1 mM EDTA, and 1 mM HADA were added to 0.1 mL of the culture, and the cells were incubated for 30 min at 30°C on a roller. The cells were then harvested by centrifugation (4000 × *g*, 2 min) and washed twice with LB. After the last wash, the cells were concentrated 10× in LB supplemented with 10 µg/mL Alexa-594-WGA and spotted on a 1% agarose pad containing LB supplemented with 10 µg/mL Alexa-594-WGA, covered with a #1.5 coverslip, and imaged as described below.

### Sample preparation for tracking LPS transport

Overnight cultures were grown in LB supplemented with 0.2% glucose and the appropriate antibiotic at 30°C. The next day, the cultures were diluted 1:100 into LB supplemented with 0.2% glucose and the appropriate antibiotic and grown at 30°C until they reached an OD_600_ of 0.2 to 0.4. The cells were then harvested by centrifugation (2 min, 5000 × *g*, RT) and washed twice with LB containing the appropriate antibiotic. After the last wash, the cells were concentrated 10× in LB containing the appropriate antibiotic, 0.2% arabinose, and 10 µg/mL Alexa-594 WGA (W11262, Thermo Fisher Scientific, MA, USA). The cells were then spotted on a 1% LB pad supplemented with 0.2% arabinose and 10 µg/mL Alexa-594 WGA, covered with a #1.5 coverslip, and imaged as described below.

### LptD localization

Overnight cultures of strain LD338 encoding an LptD-Halo fusion at the native locus (20) were grown in LB supplemented with 0.2% glucose and the appropriate antibiotic at 30°C. The next day, the cells were back diluted 1:100 into LB supplemented with the appropriate antibiotic and grown at 30°C until they reached an OD_600_ of 0.2 to 0.4. Then, the Janelia Fluor 646 Halo Tag Ligand (GA112A, Promega, WI, USA) was added to 1 mL of the culture (200 nM), and the cells were incubated for 20 min at RT on a roller. The cells were then fixed at RT for 15 min with 4% paraformaldehyde. Afterward, the cells were harvested by centrifugation (4,000 × *g*, 2 min) and washed twice with phosphate-buffered saline (PBS). After the last wash, the cells were concentrated 10× and spotted on a 1% agarose pad containing PBS, covered with a #1.5 coverslip, and imaged as described below.

Imaging was performed on a Nikon Ti2-E inverted widefield microscope equipped with a Lumencor Spectra III light engine, Semrock dichroics (LED-DA/FI/TR/Cy5/Cy7-5X-A-000), and emission filters (FF01-680/42) and recorded using a 1.45 NA Plan Apo ×100 PH3 oil objective with Olympus type F immersion oil and a pco.edge 4.2bi back-illuminated cooled sCMOS camera using Nikon Elements 5.2.

### Live-cell microscope imaging

All images were taken on a Nikon Ti-E inverted widefield microscope equipped with perfect focus system and a motorized stage. The temperature was maintained at 30°C by a custom-made environmental enclosure. Images were acquired using a Plan Apo 100×/1.45 Oil Ph3 objective lens with Cargille Type 37 immersion oil. Fluorescence was excited using a Lumencor Spectra III LED light engine and filtered using ET-GFP (Chroma, 49002), ET-DAPI (Chroma, 49000), and ET-mCherry (Chroma, 49008) filter sets as needed. Images were recorded on an Andor Zyla 4.2 Plus sCMOS camera (65  nm pixel size) using Nikon Elements (v5.10) acquisition software. For deconvolution, ten Z-planes (200 nm/step) were acquired.

### Image processing

Deconvolution was only used for images in Fig. 6. It was performed using the classical maximum likelihood estimation algorithm in Huygens Essential v19.10 (SVI). The deconvolved fluorescence images were merged with the phase-contrast images using Fiji. For all images, cropping and additional processing were performed using Fiji software. For the fluorescence images shown in the figures, the background was subtracted using the Fiji rolling ball with a radius of 50 pixels. For kymographs and the average fluorescence intensity, cells were selected using the Fiji plugin MicrobeJ. If two cells were too close together and considered as one, it was manually corrected.

## SUPPLEMENTAL MATERIAL

Supplemental Material mbio.03950-24-s0001.pdf Supplemental figures, table, and methods.

ASM does not own the copyrights to Supplemental Material that may be linked to, or accessed through, an article. The authors have granted ASM a non-exclusive, world-wide license to publish the Supplemental Material files. Please contact the corresponding author directly for reuse.
