# Supplementary material for: Using fluorescently labeled wheat germ agglutinin to track lipopolysaccharide transport to the outer membrane in Escherichia coli
Source: mBio. 2025 Feb 24;16(3):e03950-24. doi: 10.1128/mbio.03950-24 (PMC11898776; doi:10.1128/mbio.03950-24)
Supplement: Supplemental Material — Supplemental figures, table, and methods. [file mbio.03950-24-s0001.pdf]

Supplemental Material For:

Using fluorescently labeled wheat germ agglutinin to track lipopolysaccharide transport to the outer membrane in *Escherichia coli*

Laurent Dubois[1], Andrea Vettiger[1†], Jackson A. Buss[1‡], Thomas G. Bernhardt[1,2,*]

Affiliations:

[1]Department of Microbiology
Harvard Medical School
Boston, MA 02115

[2]Howard Hughes Medical Institute

*To whom correspondence should be addressed
Thomas G. Bernhardt
Harvard Medical School
Department of Microbiology
Boston, MA 02115
e-mail: thomas_bernhardt@hms.harvard.edu

[†]Present address:
Université de Lausanne
Department of Fundamental Microbiology
Batiment Biophore 2310.2
CH-1015 Lausanne

[‡]Present address:
New England Biolabs
Ipswich, MA

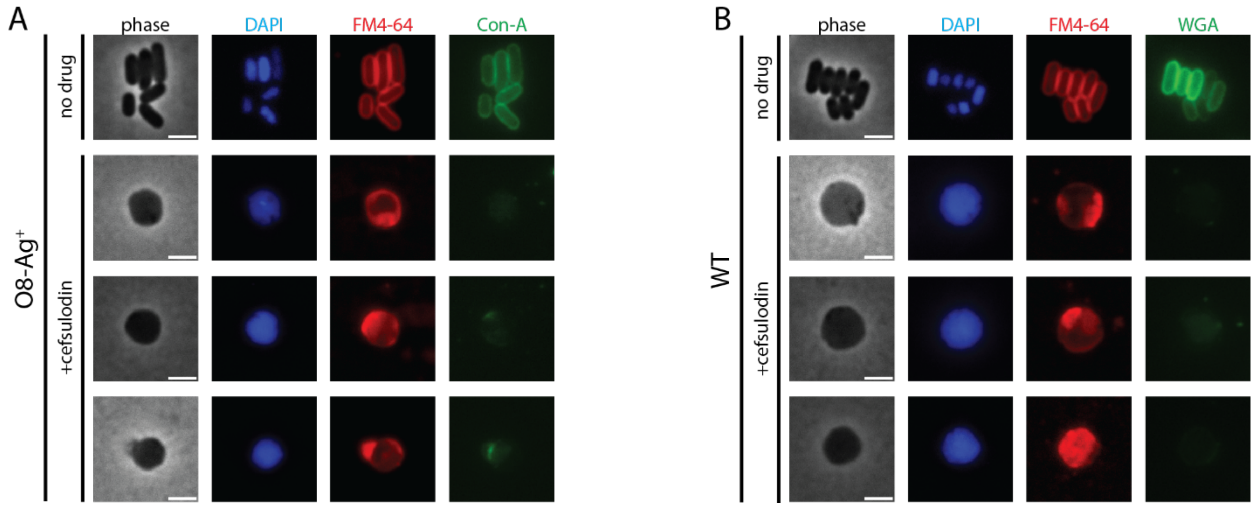

Figure S1. Additional images of L-form-like cells. Cultures of MG1655 [WT] or strain JAB595 [*rfb*O8, O8-Ag^{+}] were grown with or without cefsulodin to induce the formation of L-form-like cells. They were then stained with the indicated dyes and imaged using phase and the appropriate fluorescence optics. See Materials and Methods for details. Bar equals 2 μm.

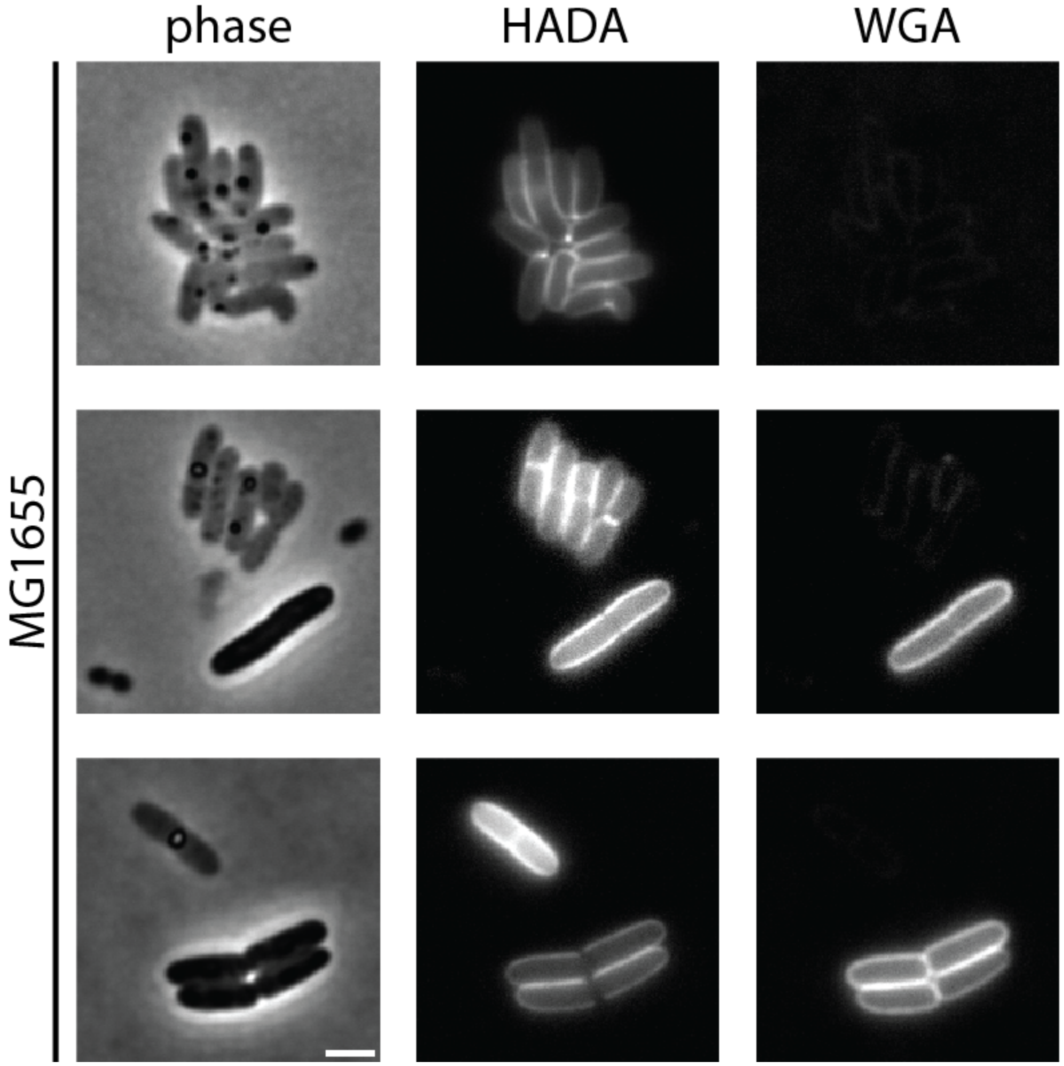

Figure S2. WGA does not bind to peptidoglycan in detergent treated cells. The outer membrane from cells of strain MG1655 was disrupted with 5% SDS and 1mM EDTA at 30°C in LB supplemented with HADA. Cells were then washed twice and imaged at 30°C on an LB pad supplemented with Fl-WGA. Lysed cells appear grey in the phase image, whereas cells that survived the treatment procedure appear phase-dark. Lysed cells do not label with FL-WGA. Bar equals 2 μm.

Bacterial Strains, Plasmids and Primers

Strain	Genotype[1]	Source/Reference[2]
Dh5a(lpir)	*F- hsdR17 deoR recA1 endA1 phoA supE44 thi-1 gyrA96 relA1 Δ(lacZYA-argF)U169 Ø80dlacZΔM15 λpir*	Laboratory strain
MG1655	rph1 IvG rfb-50	(*1*)
2443	*thr-1 leuB6 Δ(gpt-proA)66 argE3 thi-1 rfbO8lacYl ara-14 galK2 xyl-5 mtl-1 mgl-51 rpsL31 kdgK51*	(*2*)
AV52	MG1655 Δ*wecA*::frt	JAB692/pCP20
JAB595	MG1655 *rfbO8*+ *hisG*+	P1 (2443) x MG1655
JAB697	MG1655 *waaL::aph*	This study, Lambda recombineering.
JAB692	MG1655 *wecA::aph*	This study, Lambda recombineering.
JW2017	*Δ(araD-araB)567 ΔlacZ4787(::rrnB-3) rph-1 Δ(rhaD-rhaB)568 hsdR514 ΔwbbK::kan*	(*3*)
JW3763	*Δ(araD-araB)567 ΔlacZ4787(::rrnB-3) rph-1 Δ(rhaD-rhaB)568 hsdR514 ΔrffH::kan*	(*3*)
JW3765	*Δ(araD-araB)567 ΔlacZ4787(::rrnB-3) rph-1 Δ(rhaD-rhaB)568 hsdR514 ΔwecE::kan*	(*3*)
JW3770	*Δ(araD-araB)567 ΔlacZ4787(::rrnB-3) rph-1 Δ(rhaD-rhaB)568 hsdR514 ΔwecG::kan*	(*3*)
JW5596	*Δ(araD-araB)567 ΔlacZ4787(::rrnB-3) rph-1 Δ(rhaD-rhaB)568 hsdR514 ΔwecF::kan*	(*3*)
JW5599	*Δ(araD-araB)567 ΔlacZ4787(::rrnB-3) rph-1 Δ(rhaD-rhaB)568 hsdR514 ΔwecC::kan*	(*3*)
LD137	MG1655 Δ*waaL*::frt	JAB697/pCP20
LD142	MG1655 Δ*wecE::kan*	P1(JW3765) x MG1655
LD143	MG1655 Δ*wecG::kan*	P1(JW3770) x MG1655

LD144	MG1655 Δ*rffH::kan*	P1 (JW3763) x MG1655
LD146	MG1655 Δ*wecF::kan*	P1 (JW5596) x MG1655
LD149	MG1655 Δ*wecC::kan*	P1 (JW5599) x MG1655
LD159	MG1655 Δ*wecG::frt*	LD143/pCP20
LD175	MG1655 Δ*wbbK::kan*	P1(JW2017) x MG1655
LD176	MG1655 Δ*waaL::frt* Δ*wecG::kan*	P1(JW3770) x LD137
LD181	MG1655 Δ*wbbK::frt*	LD175/pCP20
LD189	MG1655 + pEMF130	MG1655/pEMF130
LD206	rph1 ivG rfb-50 Δ*wecG::frt* + pEMF130	LD159/pEMF130
LD208	MG1655 Δ*wbbK::frt* + pEMF130	LD181/pEMF130
LD214	MG1655 Δ*waaL::frt* Δ*wecG::frt*	LD176/pCP20
LD228	MG1655 Δ*waaL::frt* Δ*wecG::frt* + pLD45	LD214/pLD45
LD330	MG1655 *lptD-30aa-Halo::kan*	Kan^R version of LT18 (*4*)
LD338	rph1 IvG rfb-50 Δ*waaL::frt* Δ*wecG::frt* *lptD-30aa-Halo::kan* + pLD45	P1(LT18) x LD228
LD502	MG1655 + pLD41	MG1655/pLD41

[1] The kan cassette is flanked by frt sites. Flipping out the kan cassette by FLP recombinase, leaves an frt-scar.

[2] The P1 transductions are labeled as following: P1(donor strain) x recipient strain.

**Plasmid**	**Genotype**	**Origin**	**Source/Reference**
pCP20	cat bla cI857 $P_{\lambda R}$:FLP	pSC101(ts)	(*5*)
pEMF130	Tet^R , P_{ara}::artificalRBS_wbbL	pBR/colE1	(*6*)
pLD41	Tet^R , P_{ara}::artificalRBS_wecG	pBR/colE1	This study

pLD45	Tet^{R} , P_{ara}::nativeRBS_waaL	pBR/colE1	This study

**Primer Name**	**Sequence**	**Plasmid**
wecG-XbaI-RBS-NdeI5'	GCTATCTAGATTAAGAAGGAGATATACATATGAATAACAACACCACGGCACCAACC	pLD41
wecG_HindIII_Rev	GCTAAAGCTTTCATAGGTTGCCGGTGTAGTG	pLD41
waaL_20bp_XbaI_For	GCTATCTAGACATCATTATAAAGGTAAAACATGC	pLD45
waaL_HindIII_Rev	GCTAAAGCTTTTAATTAATTGTATTGTTACGATTATTAATGACG	pLD45

Material and Methods

Molecular Biology

The PCR were done using Q5 High-Fidelity 2x Master Mix (New England Biolabs) following manufacturer's protocol. PCR products were purified using the PCR clean up kit from Qiagen, and plasmids were isolated using the miniprep kit from Qiagen following the manufacturer's protocol.

pLD41

wecG was amplified by colony PCR from MG1655 using primers wecG-XbaI-RBS-NdeI5' (GCTATCTAGATTAAGAAGGAGATATACATATGAATAACAACACCACGGCACCAACC) and wecG_HindIII_Rev (GCTAAAGCTTTCATAGGTTGCCGGTGTAGTG). The resulting PCR product and pNP146 were digested with the restriction enzymes xbaI and hindIII and ligated using T4 ligase. The plasmid was confirmed via PCR.

pLD45

waaL was amplified by colony PCR from MG1655 using primers waaL_20bp_XbaI_For (GCTATCTAGACATCATTATAAAGGTAAAACATGC) and waaL_HindIII_Rev (GCTAAAGCTTTTAATTAATTGTATTGTTACGATTATTAATGACG). The resulting PCR product and pNP146 were digested with the restriction enzymes xbaI and hindIII and ligated using T4 ligase. The plasmid was confirmed via PCR.

1. M. S. Guyer, R. R. Reed, J. A. Steitz, K. B. Low, Identification of a Sex-factor-affinity Site in E. coli as γδ. *Cold Spring Harb Symp Quant Biol* **45**, 135–140 (1981).
2. A. S. Ghosh, K. D. Young, Helical disposition of proteins and lipopolysaccharide in the outer membrane of Escherichia coli. *J Bacteriol* **187**, 1913–1922 (2005).
3. T. Baba, T. Ara, M. Hasegawa, Y. Takai, Y. Okumura, M. Baba, K. A. Datsenko, M. Tomita, B. L. Wanner, H. Mori, Construction of Escherichia coli K-12 in-frame, single-gene knockout mutants: the Keio collection. *Mol Syst Biol* **2**, 2006.0008 (2006).
4. L. Törk, C. B. Moffatt, T. G. Bernhardt, E. C. Garner, D. Kahne, Single-molecule dynamics show a transient lipopolysaccharide transport bridge. *Nature* **623**, 814–819 (2023).
5. P. P. Cherepanov, W. Wackernagel, Gene disruption in Escherichia coli: TcR and KmR cassettes with the option of Flp-catalyzed excision of the antibiotic-resistance determinant. *Gene* **158**, 9–14 (1995).
6. E. M. Fivenson, P. DA Rohs, A. Vettiger, M. Sardis, G. Torres, A. Forchoh, T. G. Bernhardt, A role for the Gram-negative outer membrane in bacterial shape determination. *Proc Natl Acad Sci USA* **120** (2023).
